# Causal Effect of Age at Menarche on the Risk for Depression: Results From a Two-Sample Multivariable Mendelian Randomization Study

**DOI:** 10.3389/fgene.2022.918584

**Published:** 2022-07-12

**Authors:** Raphael Hirtz, Christine Hars, Roaa Naaresh, Björn-Hergen Laabs, Jochen Antel, Corinna Grasemann, Anke Hinney, Johannes Hebebrand, Triinu Peters

**Affiliations:** ^1^ Division of Pediatric Endocrinology and Diabetology, Department of Pediatrics II, University Hospital Essen, University of Duisburg-Essen, Essen, Germany; ^2^ Department of Child and Adolescent Psychiatry, Psychosomatics and Psychotherapy, University Hospital Essen, University of Duisburg-Essen, Essen, Germany; ^3^ Institute of Medical Biometry and Statistics, University Medical Center Schleswig-Holstein—Campus Lübeck, University of Lübeck, Lübeck, Germany; ^4^ Department of Pediatrics, Division of Rare Diseases and CeSER, St. Josef-Hospital, Ruhr-University Bochum, Bochum, Germany

**Keywords:** depression, age at menarche, Mendelian randomization, BMI, puberty

## Abstract

A fair number of epidemiological studies suggest that age at menarche (AAM) is associated with depression, but the reported effect sizes are small, and there is evidence of residual confounding. Moreover, previous Mendelian randomization (MR) studies to avoid inferential problems inherent to epidemiological studies have provided mixed findings. To clarify the causal relationship between age at menarche and broadly defined depression risk, we used 360 genome-wide significantly AAM-related single-nucleotide polymorphisms (SNPs) as instrumental variable and data from the latest GWAS for the broadly defined depression risk on 807,553 individuals (246,363 cases and 561,190 controls). Multiple methods to account for heterogeneity of the instrumental variable (penalized weighted median, MR Lasso, and contamination mixture method), systematic and idiosyncratic pleiotropy (MR RAPS), and horizontal pleiotropy (MR PRESSO and multivariable MR using three methods) were used. Body mass index, education attainment, and total white blood count were considered pleiotropic phenotypes in the multivariable MR analysis. In the univariable [inverse-variance weighted (IVW): OR = 0.96, 95% confidence interval = 0.94–0.98, *p* = 0.0003] and multivariable MR analysis (IVW: OR = 0.96, 95% confidence interval = 0.94–0.99, *p* = 0.007), there was a significant causal effect of AAM on depression risk. Thus, the present study supports conclusions from previous epidemiological studies implicating AAM in depression without the pitfalls of residual confounding and reverse causation. Considering the adverse consequences of an earlier AAM on mental health, this finding should foster efforts to address risk factors that promote an earlier AAM.

## 1 Introduction

There is a steep increase in depressive symptoms and major depressive disorder (MDD) during adolescence. Moreover, a gender gap in the prevalence of MDD emerges only by puberty, affecting twice as many girls as boys. This finding persists throughout much of adult life (Thapar et al., 2012). These observations highlight the importance of puberty on mental health, especially in pubescent girls. To better understand puberty-related mental health trajectories in girls, age at menarche (AAM) has been extensively studied.

Earlier menarche relates not only to a higher risk for externalizing problems, among others, early or risky sexual behavior (Ullsperger and Nikolas, 2017), substance use (Ullsperger and Nikolas, 2017), and peer victimization (Smith-Woolley et al., 2017) but also to internalizing problems, including anxiety (Platt et al., 2017; Smith-Woolley et al., 2017), eating disorders (Smith-Woolley et al., 2017; Ullsperger and Nikolas, 2017), and depressive symptoms (Ullsperger and Nikolas, 2017). However, effect sizes regarding internalizing outcomes are small (d ≈ 0.2) (Ullsperger and Nikolas, 2017), and there are also contradictory findings from well-designed studies (Carter et al., 2011; Gaysina et al., 2015; Winer et al., 2016), which might indicate a variable degree of confounding. Consistent with this, a more recent study by Vaughan et al. (2015), that examined the impact of AAM on depressive symptoms in adolescence by a multilevel familial design, concluded that the effects of AAM might be driven by unmeasured or residual confounding. Thus, AAM may instead act sensitizing rather than causally in an environment of endogenous and exogenous pubertal stressors to favor the development of depression (Skoog and Stattin, 2014).

However, the approach chosen by Vaughan et al. (2015) relied on several key assumptions, some of which were not tested or may not hold in the studied context. Moreover, observational studies are not only prone to residual confounding but also reverse causation (Haycock et al., 2016; Besser et al., 2021). In contrast, Mendelian randomization (MR) uses genetic markers to draw causal conclusions on the association between an exposure (e.g., AAM) and an outcome (e.g., depression) of interest by exploiting the fact that genotypes are not generally associated with confounders in the population and randomly assigned at conception, analogous to randomization in clinical trials. Moreover, since the individual genotype is determined upon conception and cannot be modified by the outcome of interest, MR is robust to reverse causation. Usually, single-nucleotide polymorphisms (SNPs) derived from large-scale genome-wide association studies (GWASs) are used as instrumental variables (IVs). While in the one-sample MR setting, the individual participant data are used to assess the causal effect of the exposure on the outcome of interest, two-sample MR studies rely on the summary statistics from independent GWAS. The latter approach allows for greater sample sizes and, thereby, statistical power and comes with the advantage of less bias in the presence of weak genetic instruments (Haycock et al., 2016; Davies et al., 2018). However, for valid causal conclusions, MR relies on several assumptions. A potential threat to the validity of MR findings is horizontal pleiotropy, which refers to an effect of the IV on the outcome (depression) by a mechanism other than *via* the exposure (AAM). Previously, BMI has been identified as a potential pathway to horizontal pleiotropy when using AAM as exposure as a large number of SNPs associated with AAM are also negatively related to BMI (Day et al., 2017). When also considering that a higher BMI is associated with a higher risk for depression (Casanova et al., 2021), BMI is a potential source of horizontal pleiotropy. Moreover, recently, Magnus et al. (2020) have shown in their one-sample MR phenome-wide association study with 17,893 health-related traits in the UK Biobank that many phenotype categories, such as sociodemographic characteristics, substance use and addictions, mental health and traumatic events, measures of cognition and aging, blood parameters, and others, might also be relevant in this regard. Thus, MR studies addressing the causal relationship between AAM and MDD also have to be judged by the degree to which horizontal pleiotropy is considered. For example, a one-sample MR study of 12.233 Chinese women aged 50 years and older could not find evidence of a significant contribution of AAM to depressive symptoms in older women (Au Yeung et al., 2018). However, despite no apparent evidence of a weak instrument bias, their analysis included only five AAM-related SNPs and did not account for BMI. In contrast to the null finding of this study, Sequeira et al. (2017) demonstrated an effect of AAM on depression in 3.579 longitudinally studied girls at the age of 14, but not thereafter. This study not only included 120 AAM-related SNPs but also accounted for BMI. However, as recently discussed, this analysis was likely to be affected by low power (Chan et al., 2019). This conclusion is supported by the results from a recent large-scale, one-sample MR study of 181,318 women from the UK Biobank that could relate AAM to a broad, categorical depression phenotype in adult women (40–69 years) (Magnus et al., 2020). Notably, apart from a (much) larger sample size, the study was based on a more recent and extended set of 360 AAM-related SNPs but did not consider other potential causes of horizontal pleiotropy in addition to BMI. Regarding two-sample MR, a single, more recent study found no evidence of a causal relationship between AAM and depression (Howard et al., 2019), but the study included only 60 genetic variants for methodological reasons.

Considering this somewhat inconsistent picture, the present study was intended to clarify the directional relationship between AAM and risk for depression by 1) a two-sample Mendelian randomization approach with the so-far largest sample size and 2) considering BMI and other potential causes of horizontal pleiotropy on this relationship by a multivariable MR (MVMR) analysis. Establishing this causal link would not only support paying particular attention to the development of mental health in early maturing girls but also justify addressing risk factors that promote experiencing menarche at an earlier age.

## 2 Methods and Materials

### 2.1 Univariate Mendelian Randomization Analysis

To perform an MR study, three main assumptions must be met (Lawlor et al., 2008; König and Greco, 2018): 1) the genetic instrument must have a strong association with the exposure, 2) the genetic instrument is independent of potential confounding factors in the relationship between the exposure and outcome, and 3) the outcome is associated with the genetic instrument only through the effect of the exposure (Haycock et al., 2016; König and Greco, 2018).

Only the first assumption can be tested directly by assessing the F-statistic (Haycock et al., 2016). An F < 10 suggests potential weak instrument bias (Sanderson et al., 2020). Independent genome-wide significant SNPs (*p* < 5 × 10^–8^) were used as IV for AAM to avoid such a bias. The second assumption is unlikely to be violated in the MR context as genetic variants are fixed at conception and cannot be influenced by the confounding factors of the risk factor–outcome association (Del Greco et al., 2015). To assess the third assumption, we used different approaches. First, we used Cochran’s Q-statistic to test for IV heterogeneity, which may have several causes, of which horizontal pleiotropy is most likely. Cochran’s Q-statistic assesses whether causal estimates of SNPs are comparable (Bowden et al., 2015). In the univariate MR case, a significant finding (*p* < 0.05) indicates heterogeneity. Second, to specifically address horizontal pleiotropy, we calculated MR–Egger regression and performed MR-PRESSO analysis. If Egger’s intercept is not significantly different from zero, horizontal pleiotropy is unlikely (Burgess and Thompson, 2017). MR PRESSO uses a global bias test to evaluate whether the removal of potentially pleiotropic instruments results in a significant difference in the overall causal estimate and provides a corrected causal estimate after the removal of pleiotropic instruments. Comparing both the approaches, simulation studies have shown that MR PRESSO is more sensitive to horizontal pleiotropy than Egger’s intercept (Verbanck et al., 2018).

It is unlikely that all IVs meet the instrumental variable assumptions. Therefore, several MR methods have been developed, which differ in their robustness to various violations of assumptions. Considering that no method alone provides an infallible test of causality, the use of different methods to assess whether a causal effect determined by MR is robust has been recommended (Burgess et al., 2017a; Burgess et al., 2019). Accordingly, we applied the following MR methods: 1) the inverse-variance weighted (IVW) method assumes that all ratio estimates provide independent evidence of the causal effect and that all genetic variants are valid instruments. No intercept term is included in the regression model (Burgess and Thompson, 2017); 2) MR–Egger considers an intercept term interpreted as the average pleiotropic effect of the genetic variants included in the analyses. If the pleiotropic effects are distributed independently of the genetic associations with the risk factor (InSIDE assumption: INstrument Strength Independent of Direct Effect), the MR–Egger estimate is a consistent estimate of the causal effect as both sample size and the number of genetic variants increase (Burgess and Thompson, 2017; Hartwig et al., 2017); 3) Mode-based estimation (MBE; simple mode, weighted mode) consistently estimates the true causal effect under the assumption that across all instruments, the most frequent value of bias due to pleiotropy is 0 [ZEro-modal pleiotropy assumption (ZEMPA)] (Hartwig et al., 2017). Simple MBE is less accurate than weighted MBE, but simple MBE is less prone to bias due to violations of the InSIDE assumption; 4) median-based estimators are consistent even when up to 50% of the instruments are invalid. The weighted median estimator has similar efficiency to the IVW method, but the simple median estimator is less efficient than either the IVW or the weighted median method (Bowden et al., 2016). The penalized weighted median estimator is robust in the case of IV heterogeneity (Rees et al., 2019); 5) The robust-adjusted profile score (MR RAPS) provides an overall estimator that is robust to systematic and idiosyncratic pleiotropy (some genetic instruments have a large effect on the outcome) (Zhao et al., 2020); 6) MR PRESSO (see above) (Verbanck et al., 2018).

In the case of IV heterogeneity, as indicated by Cochran’s Q statistic, two methods robust to heterogeneity were used in addition to the penalized weighted median method (Rees et al., 2019): 7) the contamination mixture method has a good overall performance (the lowest mean squared error), with up to 40% invalid instruments compared to other robust methods. The contamination mixture method identifies distinct subgroups of genetic variants with mutually similar causal estimates (Burgess et al., 2020); 8) MR-Lasso extends the IVW model to include an intercept term for each genetic variant. These intercept terms represent associations between genetic variants and the outcome that bypasses the risk factor. The causal effect is estimated by weighted linear regression, with the intercept terms subjected to lasso penalization. Lasso penalization shrinks the intercept of the valid instruments to zero (Rees et al., 2019).

Forest and scatter plots were used to visualize the combined results of single and multi-SNP analyses. The scatter plots show single SNP effects on the exposure against single SNP effects on the outcome with corresponding standard deviations and estimated regression lines of the multi-SNP analyses. We created funnel plots to examine the relationship between study accuracy and effect size (Haycock et al., 2016). Asymmetry in the funnel plot indicates a violation of MR assumptions (Katikireddi et al., 2018). To assess whether a single SNP had a disproportionately large effect on the regression coefficients, we performed a leave-one-out analysis in the IVW regression framework (Burgess and Thompson, 2017).

Power analysis to estimate the probability of finding a true effect was implemented using sample size, the proportion of cases in the study, and the proportion of variance explained (SNP heritability) (Brion et al., 2013).

### 2.2 Multivariable Mendelian Randomization Analysis—Assessing the Effect of BMI and Other Causes of Pleiotropy

An association of BMI with AAM and depression has been established by previous research (Day et al., 2017; Magnus et al., 2020). To identify other potential causes of horizontal pleiotropy, we submitted all the SNPs associated with AAM (*n* = 343) to the PhenoScanner database (Staley et al., 2016; Kamat et al., 2019) to establish significant phenotypical associations with these SNPs (*p* < 1 × 10^–5^) in Europeans. Thereby, and by an additional review of the literature including GWASs on the relationship between identified phenotypes and MDD, we also included educational attainment (EdAtt) and white blood cell count (WBC) for MVMR analyses as covariates.

To estimate the effects of these pleiotropic phenotypes in the association between AAM and depression, we performed MVMR using three different methods (Burgess and Thompson, 2015): 1) the inverse-variance method uses a multivariable weighted linear regression; 2) multivariable MR-Lasso extends the multivariable IVW model to include an intercept term for each genetic variant; and 3) the multivariable median method is similar to the univariable weighted median method, except that it relies on quantile regression (Burgess and Thompson, 2015).

To test the IV strength, we calculated the conditional F-statistics F_TS_. Furthermore, we calculated the Q-statistic to test for heterogeneity. In contrast to the univariate case, a *p*-value < 0.05 indicates that excessive heterogeneity in the MVMR model can be rejected and that the selected SNPs can predict the exposure phenotypes (Sanderson et al., 2020). We could not consider correlations between exposure phenotypes because individual-level genetic data were not available to us.

### 2.3 Sensitivity Analyses

We excluded SNPs that were strongly associated with potentially pleiotropic phenotypes from the univariable MR analysis addressing the relationship between AAM and depression risk. For this purpose, we calculated an F-statistic using the formula F = (beta/se)^2^ for each SNP defining the IV for AAM separately for BMI, EdAtt, and WBC. In line with previous studies (Swerdlow et al., 2016; Burgess et al., 2017b), we excluded SNPs with F > 10 from the analysis, assuming that these SNPs are also strong instruments for potentially pleiotropic phenotypes.

### 2.4 Data Sources for Mendelian Randomization Analyses and Selection of the Genetic Instruments

#### 2.4.1 Age at Menarche

We performed MR analyses based on the GWAS by Day et al. (2017), which comprised 40 studies from the ReproGen consortium, 23andMe, and the UK Biobank. The data from 329,345 women of European ancestry were included. The women were asked to report when the first menstruation/period (menarche) occurred. The regression model included age at study visit and other study-specific covariates. There were 389 independent SNPs (*p* < 5 × 10^–8^) associated with AAM. The per-allele effect sizes were expressed in years and ranged from ∼1 week to 5 months. Depending on the study, these 389 independent SNPs explained 7.2–7.4% of the trait variance (Day et al., 2017). The total chip-captured SNP-based heritability (h^2^_SNP_) for AAM estimated on the UK Biobank data was 32%.

#### 2.4.2 Depression

We used the latest GWAS of 807,553 individuals (246,363 cases and 561,190 controls) by Howard et al. (2019) as data source for depression as the outcome variable. The authors meta-analyzed the data from the three largest genome-wide association studies of depression. The phenotyping of depression differed in these three studies: 1) self-reported clinical diagnosis of depression by Hyde et al. (2016); 2) spectrum of depression phenotypes obtained from a structured clinical interview or based on broader criteria by Wray et al. (2018); and 3) self-reported help-seeking for problems with nerves, anxiety, tension, or depression (broad depression) by Howard et al. (2018). The overlapping samples were excluded. Altogether, 102 independent variants were identified. The proportion of women in the GWASs was found to be 48% by Hyde et al. (2016) and 54% by Howard et al. (2018). The proportion of males and females was not reported by Wray et al. (2018). The regression model on the UK Biobank data included sex, age, genotyping array, and the first eight principal components for population structure (Howard et al., 2019). The regression model on the 23andMe data included the covariate age, sex, and the top five principal components (Hyde et al., 2016). The genome-wide SNP-based heritability (h^2^_SNP_) was 8.9% (Howard et al., 2019).

#### 2.4.3 BMI

We used the most recent GWAS on the BMI, where separate analyses were performed for sexes (Pulit et al., 2019). A total of 806,834 individuals of European ancestry were included in the analysis, of which 434,794 were females. The SNP-based heritability (h^2^_SNP_) for females was 35.5% for all the SNPs and 30.0% for SNP, with a mean allele frequency below 1% (Pulit et al., 2019). The covariates included in the regression model for BMI are not reported by the authors.

#### 2.4.4 Educational Attainment

To our knowledge, the most recent and largest GWAS on EdAtt was conducted by Lee et al. (2018). This analysis was based on data from 1,131,881 individuals of European ancestry and identified 1,271 independent genome-wide significant SNPs. The SNP-based heritability was 12.2%. EdAtt was measured at the age of at least 30 by the number of years of schooling completed. The phenotype was constructed by mapping each major educational qualification that can be identified from the survey measure of the cohort to an International Standard Classification of Education (ISCED) category and imputing years-of-education equivalent for each ISCED category (Lee et al., 2018). Across all cohorts, the sample-size-weighted mean of educational years was 16.8 (SD = 4.2) of schooling. The following covariates were considered in the regression model: first ten principal components, standardized controls, including a third-order polynomial for the year of birth, sex, and their interactions, and a vector of study-specific controls.

#### 2.4.5 Total White Blood Cell Count

We used the data from the latest GWAS on blood parameters for which summary statistics were made available (Chen et al., 2020). We used the results on the WBC of 562,132 participants of European descent. Information on the proportion of men and women is not published. The common SNPs explained 19% of the variance (h^2^_SNP_). WBCs were measured at a scale of 10^–9^ cells/L. The blood-cell phenotypes were corrected for sex, age, age squared, the first 10 genetic principal components, and other cohort-specific covariates (e.g., recruitment center) using the linear regression analysis. A rank-based inverse normal transformation of the residuals from the regression analysis was applied.

### 2.5 Statistical Analysis

Analyses were performed using the software “R” (3.5.1 and 4.1.1) and the R packages “TwoSampleMR” (0.4.26; https://github.com/MRCIEU/TwoSampleMR) (Hemani et al., 2018), “Mendelian Randomization” (0.5.1; https://CRAN.R-project.org/package=MendelianRandomization), “MVMR” (0.3; https://github.com/WSpiller/MVMR) (Sanderson et al., 2020), and “MR Practicals” (0.0.1; https://github.com/WSpiller/MRPracticals). MR PRESSO was conducted with the R package “rondolab/MR-PRESSO” (1.0; https://github.com/rondolab/MR-PRESSO) (Verbanck et al., 2018).

We followed the “STROBE-MR: Guidelines for strengthening the reporting of Mendelian randomization studies for the two-sample MR” recommendations reporting our results (Skrivankova et al., 2021).

## 3 Results

### 3.1 Univariable Mendelian Randomization

**FIGURE 1:**
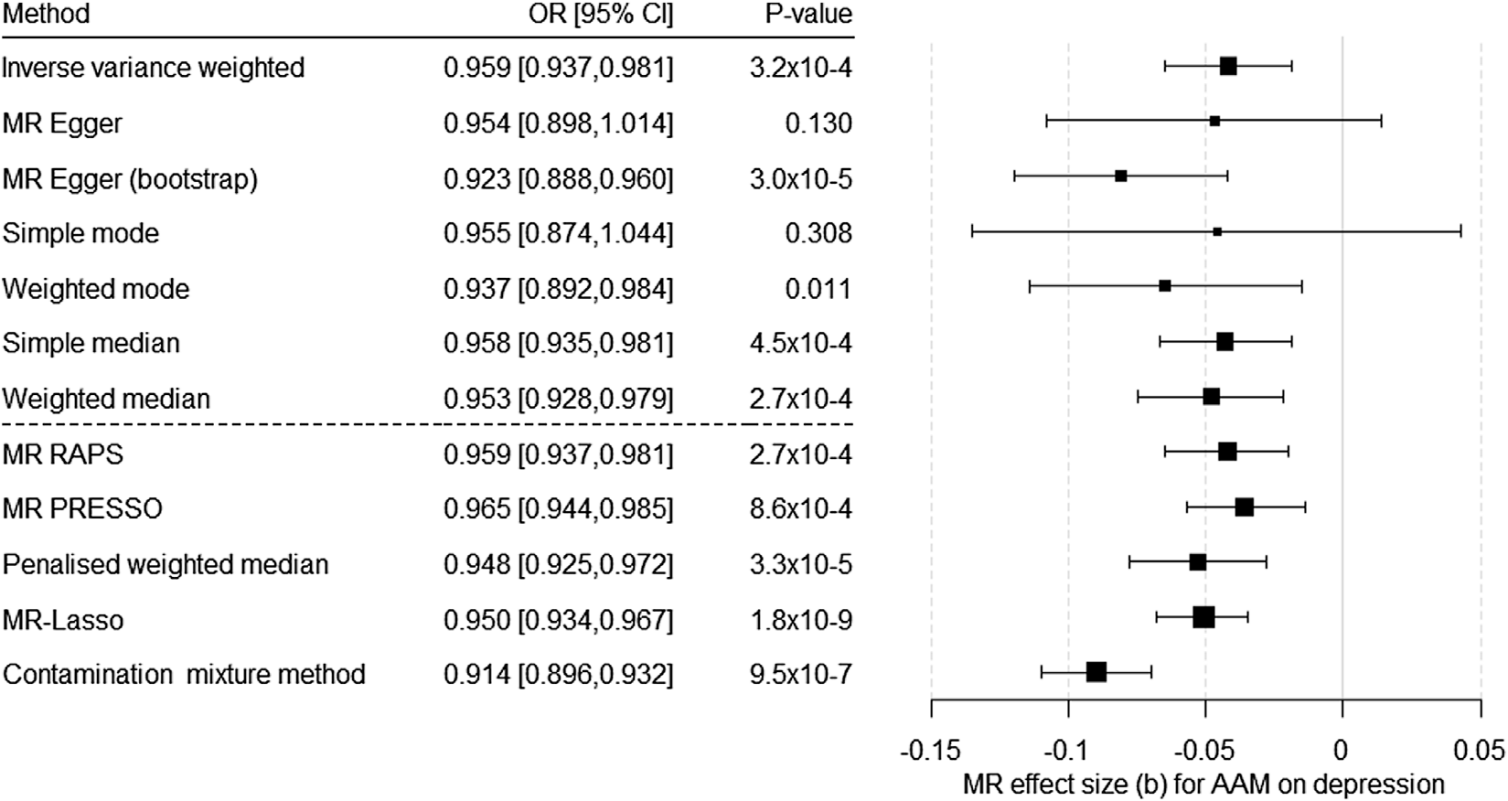
Results of the multiple-SNP Mendelian randomization (MR) analyses regarding the effect of age at menarche (AAM) on the risk for depression. OR = odds ratio, CI = confidence interval, b = unstandardized causal estimate of the change in risk for depression per 1-year change in age of menarche.

In the analysis on the causal effect of AAM on the risk for depression, 343 SNPs could be included as IV for AAM (for the harmonized data, see Supplementary Table S4. The F-test indicated a strong IV (F = 62,655.97). We found a significant causal effect of AAM on the risk for depression with almost all methods (except MR–Egger without bootstrapping and simple mode; Figure 1; Supplementary Table S1). Egger’s intercept was not significant (intercept = 2.1 × 10^–4^, SE = 0.001, *p* = 0.85). In contrast, MR PRESSO detected evidence of pleiotropy (global test 840.45, *p* = 1 × 10^–4^). In addition, the heterogeneity test indicated a potential problem (based on MR Egger: Q (df = 341) = 835.13, *p* = 2.22 × 10^–42^; based on IVW Q (df = 342) = 835.21, *p* = 3.49 × 10^−43^), but all the methods robust to heterogeneity (penalized weighted median, MR Lasso, and contamination mixture method) showed a significant causal effect of AAM on the risk for depression (Figure 1; Supplementary Table S1). The scatter plot, funnel plot, and forest plot for the leave-one-out analysis indicated no violation of MR assumptions or an undue impact of single SNPs on the results (Supplementary Figures S1–S3). Power analysis showed that our MR had a power of 80% to detect an OR below 0.975 or above 1.026 and a power of 100% to detect an OR below 0.955 or above 1.043 regarding depression per one-year change in AAM (Supplementary Figure S4).

### 3.2 Multivariable Mendelian Randomization: Effect of AAM on Depression Adjusted for BMI, Educational Attainment, and Total White Blood Cell Count

By searching the PhenoScanner database for SNPs associated with AAM (Staley et al., 2016; Kamat et al., 2019), we identified 25 SNPs associated with different measures of intelligence, EdAtt, or both. Five SNPs (rs11210871, rs11209943, rs15092139, rs1054442, and rs1131017) were associated with different measures of intelligence and EdAtt. Five SNPs were associated only with intelligence but not with EdAtt, and there were 15 SNPs associated only with EdAtt. However, considering the SNP-based overlap between intelligence and EdAtt, the larger number of EdAtt-related SNPs, and the broader conception of EdAtt, also including aspects of socioeconomic status (Thomson, 2018), EdAtt was chosen for further MVMR analysis.

Strikingly often (N = 25), AAM-related SNPs were associated with different fractions of leukocytes (neutrophils, lymphocytes, monocytes, basophils, and eosinophils, but also the total WBC). Based on an additional literature review, we decided that EdAtt (Magnus et al., 2020; Yuan et al., 2021) and WBC (Magnus et al., 2020; Sealock et al., 2021) also needed consideration in our MVMR model, while other phenotypes, such as alcohol intake and smoking, were considered downstream outcomes of AAM (Hingson et al., 2006; Barrington-Trimis et al., 2020).

**FIGURE 2:**
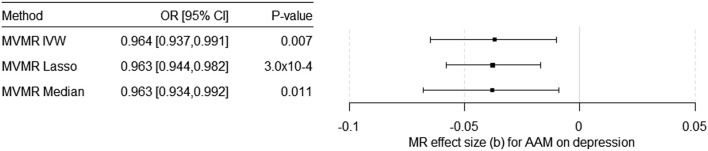
Results of the multivariable MR (MVMR) analyses of the causal effect of age at menarche (AAM) on the risk for depression adjusted for BMI in females, educational attainment, and total white blood cell count calculated using three different methods. OR = odds ratio, CI = confidence interval, b = unstandardized causal estimate of the change in risk for depression per 1-year change in age of menarche.

Considering 318 SNPs as IV for AAM and for BMI, EdAtt, and WBC as potential pleiotropic phenotypes (Supplementary Table S5) in the multivariable MR (MVMR), we found a significant causal effect of AAM on the risk for depression independent of BMI, EdAtt, and WBC (Table 1; Figure 2). The effect estimate for EdAtt, as covariable in the model on the effect of AAM on depression risk, was significant with all the three MVMR methods, whereas the effect estimate for the BMI was not significant. The effect estimate for WBC was significant only with the multivariable IVW method. The Q-statistic confirmed the validity of the IV [Q (df = 313) = 737.45; *p* = 2.8 × 10^–36^] as there was no evidence of significant heterogeneity. The conditional F-test showed that the IV was sufficiently strong for AAM (F_TS_ = 20.39) but not for BMI (F_TS_ = 9.23), EdAtt (F_TS_ = 5.36), or WBC (F_TS_ = 6.30).

**TABLE 1:** Results of the multivariable MR (MVMR) analyses of the causal effect of age at menarche on the risk for depression adjusted for BMI in females, educational attainment, and total white blood cell count calculated using three different methods.

Method	B			SE	*p*-value
	Point estimate	Lower 95% CI	Upper 95% CI		
		Age at menarche			
MVMR IVW	−0.037	−0.065	−0.010	0.014	**7 × 10** ^**–3**^
MVMR lasso^a^	−0.038	−0.058	−0.017	0.010	**3.0 × 10** ^**–4**^
MVMR median^b^	−0.038	−0.068	−0.009	0.015	**0.011**
	BMI in females				
MVMR IVW	0.048	−0.047	0.143	0.048	0.320
MVMR lasso^a^	0.039	−0.029	0.107	0.035	0.263
MVMR median^b^	0.033	−0.066	0.132	0.050	0.544
	Educational attainment				
MVMR IVW	−0.202	−0.385	−0.019	0.093	**0.031**
MVMR lasso^a^	−0.285	−0.423	−0.146	0.071	**5.6 × 10** ^**–5**^
MVMR median^b^	−0.387	−0.582	−0.183	0.099	**9.7 × 10** ^**–5**^
	Total white blood cell count				
MVMR IVW	0.177	0.022	0.333	0.079	**0.026**
MVMR lasso^a^	0.106	−0.033	0.246	0.071	0.135
MVMR median^b^	0.077	−0.099	0.252	0.090	0.394

b = unstandardized causal estimate of the change in risk for depression per 1-year change in age of menarch. SE = standard error; CI = confidence interval. Significant findings are printed in Significant findings (p < 0.05) are printed in bold type..

a Number of variants: 318, number of valid instruments: 244.

b Iterations = 10,000; tuning parameter: 0.1014.

### 3.3 Sensitivity Analyses

#### 3.3.1 Excluding SNPs Associated With BMI

**FIGURE 3:**
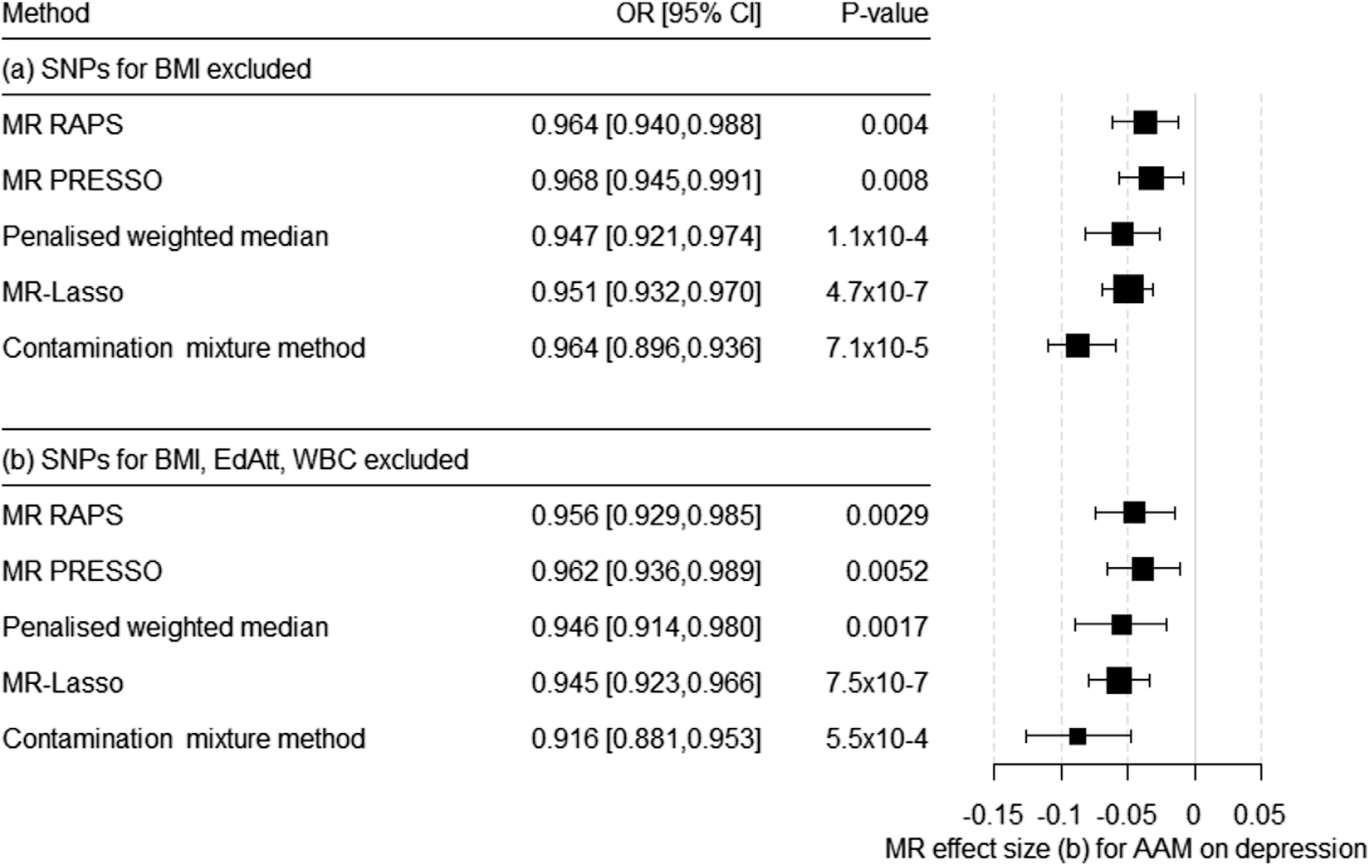
Results of the multiple-SNP Mendelian randomization (MR) analysis with robust methods regarding the effect of age at menarche (AAM) on the risk for depression. (a) SNPs associated with BMI were excluded (IV with *n* = 263 SNPs), (b) SNPs associated with BMI, educational attainment (EdAtt), and white blood cell count (WBC) were excluded (IV with *n* = 185 SNPs). OR = odds ratio, CI = confidence interval, b = unstandardized causal estimate of the change in risk for depression per 1-year change in age of menarche.

BMI was not a strong instrument in the MVMR, but previous studies have convincingly shown that BMI is, nevertheless, a potential cause of horizontal pleiotropy in the association between AAM and the risk for depression. Therefore, we conducted a sensitivity analysis by excluding all the BMI-related SNPs with F > 10 from the IV. Thus, the IV could be built using 263 SNPs (for harmonized data, see Supplementary Table S6). Egger’s intercept (= 5.8 × 10^–4^, SE = 0.001) was not significant (*p* = 0.644). In contrast, MR-PRESSO detected SNPs with pleiotropic effects (global test = 560.14, *p* = 1 × 10^–5^). Consistent with the MR-PRESSO results, heterogeneity continued to be a potential problem [Cochran’s Q-statistic based on MR–Egger: Q (df = 261) = 554.70, *p* = 2.7 × 10^–23^; based on IVW: Q (df = 262) = 555.15, *p* = 3.6 × 10^–23^]. The scatter plot, funnel plot, and forest plot for the leave-one-out analysis showed no violation of the MR assumptions or the excessive influence of individual SNPs on the results (Supplementary Figures S5–S7). The robust methods confirmed the causal effect of AAM on the risk for depression (Figure 3A; Supplementary Table S2).

#### 3.3.2 Excluding SNPs Associated With BMI, Educational Attainment, and Total White Blood Cell Count

Since educational attainment and WBC were also weak instruments in the MVMR, we conducted an additional univariate MR. Here, we excluded all the SNPs that were strong instruments for BMI, EdAtt, and/or WBC (F > 10). Thus, we were able to include 185 SNPs as IV for AAM (Supplementary Table S7). Egger’s intercept was not significant (= 3.0 × 10^–5^, *p* = 0.984). However, MR PRESSO identified SNPs with a pleiotropic effect (global test 347.18, *p* = 1 × 10^–5^). The heterogeneity tests were significant: based on MR-Egger—Q (df = 183) = 343.6, *p* = 6.8 × 10^–12^ and based on IVW—Q (df = 184) = 343.6, *p* = 9.3 × 10^−12^. Again, all robust methods suggested a significant causal effect of AAM on the risk for depression (Figure 3B; Supplementary Tables S3, Supplementary Figures S8–S10).

Eventually, pleiotropic SNPs identified by MR PRESSO by the previous step of analysis (rs10933, rs12571664, and rs2546959) were subjected to the PhenoScanner database to assess which other phenotypes, apart from BMI (or other body composition phenotypes), EdAtt, and WBC, might be causes of pleiotropy. The SNP rs10933 is located in a region associated (*p* < 1 × 10–5) with schizophrenia (Ripke, 2014), bipolar disorder (Sklar, 2011), intelligence, and some hematological traits (Astle et al., 2016). The region around SNP rs2546959 is associated with the “seen doctor for nerves, anxiety, tension, or depression”-phenotype detected by the Neale Lab (UK Biobank; http://www.nealelab.is/uk-biobank). According to the PhenoScanner database, the region around rs12571664 only showed associations with AAM and body size. Thus, there was no compelling evidence of phenotypes to pursue by further analyses.

## 4 Discussion

Observational studies suggest a robust contribution of AAM to depression, but there is also evidence of (residual) confounding. MR studies allow for causal conclusions, but findings in this regard have been ambiguous, primarily related to power issues in the previous MR analyses. In the present two-sample MR study with sufficient power to detect even small effects, an earlier AAM proved to be causal for an increased risk for depression, independent of the pleiotropic phenotypes BMI, EdAtt, and WBC, even when using multiple methods to account for heterogeneity of the IV (penalized weighted median, MR Lasso, and contamination mixture method), systematic and idiosyncratic pleiotropy (MR RAPS), and horizontal pleiotropy (MR PRESSO, exclusion of SNPs showing a relevant association with the potentially pleiotropic phenotypes, and MVMR).

### 4.1 Age at Menarche and Depression—Increased Lifetime Risk

Considering that no GWASs on depression in different age classes (e.g., children, adolescents, and adults) are available but the MR approach assesses the cumulative lifetime effects of an exposure on an outcome (Lawlor, 2016), two assumptions must be met for our conclusions to hold. First, the genetic architecture of depression, and second, the pathway(s) from AAM to depression should not change throughout the life course.

Regarding the first assumption, a meta-analysis on the heritability of depression from childhood to young adulthood found an increase in its heritability during adolescence (Bergen et al., 2007). This is in line with a more recent twin study including almost 50,000 twin pairs studied longitudinally from ages 3 to 63 (Nivard et al., 2015). However, a change in the heritability of depression does not necessarily imply a different regulation of its (genetic) underpinnings. Instead, the results from twin and adoption studies on the relative contribution of genetic and environmental influences on the heritability of depression over time suggest that this observation is likely explained within the framework of active genome-environment correlation, resulting in more opportunities during adolescence to express genetically determined dispositions, a decrease in shared environmental variance, a combination of both, and/or a reduction of measurement error related to more reliable information provided by more mature subjects (Silberg et al., 1999; Bergen et al., 2007; Nivard et al., 2015). Moreover, it has been suggested that phenotypical continuity regarding depression is mainly driven by genetic factors (Nivard et al., 2015). Consistent with this, a genome-wide association meta-analysis addressing internalizing symptoms suggested not only that (additive) genetic effects appear to be stable from early childhood to adolescence by overlapping SNP-based heritability estimates over age but also a high genetic correlation between childhood internalizing symptoms and adult depression (*r*_*g*_ > 0.7) (Jami et al., 2022). The latter finding of a high but not perfect genetic correlation could be related to observing different (genetic) depression trajectories during earlier life. Rice et al. (2019) identified two trajectories of depressive symptoms from childhood to early adulthood, one commencing in later, one in earlier adolescence. However, only late-adolescent depression was related to a polygenic MDD risk score, while early-adolescent depression was linked to a higher (genetic) ADHD and schizophrenia risk. Thus, part of the phenotypical variance in depression during childhood and adolescence seems to be associated with different genetic pathways, even though the pathway leading to increased ADHD and schizophrenia is less common (Rice et al. 2019).

Regarding the second assumption, the finding of the one-sample MR study by Sequeira et al. (2017) showed that AAM only affects depression at age 14 but not thereafter, which could be interpreted to indicate that the relationship between AAM and depression is less stable than implied by our results. This would be consistent with the observation that the severity of depressive symptoms returned to early pubertal baseline levels in young adults in a large longitudinal study of (up to) 14,500 boys and girls (Natsuaki et al., 2009). However, as previously discussed, the study by Sequeira et al. (2017) was likely affected by insufficient power at depression assessments after age 14. Moreover, a plethora of longitudinal studies suggest that depressive symptoms peak not until (mid to) late adolescence and do not return to prepubertal or early pubertal baseline levels, (Harlow et al., 1999; Mendle et al., 2018; Copeland et al., 2019; Kwong et al., 2019), and this also applies regarding the effect of early menarche on depression (Mendle et al., 2018; Copeland et al., 2019).

### 4.2 Pathways From Age at Menarche to Depression

We observed significant heterogeneity in the causal estimates of our IV. Day et al. (2017) identified 389 independent signals for AAM, many of which are implicated in a complex and hierarchical network of genes governing the onset of puberty (Lomniczi et al., 2013). However, these SNPs also related to potentially pleiotropic phenotypes in the relationship between AAM and depression, including BMI, EdAtt, and WBC. BMI and AAM are closely intertwined (Day et al., 2017), as metabolic cues originating from adipose tissue essentially control the reactivation of the reproductive axis during puberty (Vazquez et al., 2019) and BMI also relates to depression (Bahrami et al., 2020). Moreover, the PhenoScanner search revealed that EdAtt and WBC are related to AAM, and both have been implicated in the etiology of depression (Sealock et al., 2021; Yuan et al., 2021), rendering these phenotypes likely causes of horizontal pleiotropy in the relationship between AAM and depression. Nevertheless, even when accounting for potential horizontal pleiotropy introduced by these phenotypes, there was a causal effect of AAM on the risk for depression, consistent across different approaches.

Interestingly, previous GWAS on the genetic underpinnings of AAM did not identify SNPs related to the biologically most plausible pathway from AAM to depression, that is, gonadal hormone levels. There is suggestive evidence for an impact of estradiol on depressive symptoms, not only from animal studies but also studies in pubescent adolescents (Brooks-Gunn and Warren, 1989; Ge and Natsuaki, 2009; Schulz et al., 2009). However, consistent with findings from GWASs, there are also conflicting results from observational studies (Golub and Harrington, 1981; Copeland et al., 2019; Morssinkhof et al., 2020), and a more recent one-sample MR study does not support a causal effect of estrogen levels on depressive symptoms (Au Yeung et al., 2016). Moreover, neither is there evidence of increased androgen levels in female MDD patients (de Wit et al., 2021) nor are testosterone levels in females likely causally related to depression (Maharjan et al., 2021). Whether upstream mechanisms of estrogen secretion are important in this regard remains to be determined but cannot be excluded. For example, LH receptors are expressed not only in testicular and ovarian tissue but also in the hippocampus (Bohm-Levine et al., 2020), a brain region pivotal to depression (Campbell and Macqueen, 2004). Moreover, depression is marked by altered LH secretion (Grambsch et al., 2004). However, altered pulsatile LH release from the pituitary gland might also be the consequence of increased cortisol levels in depression (Breen et al., 2005; Zorn et al., 2017) and, thus, simply an epiphenomenon of MDD but not its cause.

However, only recently, Magnus et al. (2020) opened up a perspective on the pathways from AAM to depression by a (one-sample) Mendelian randomization phenome-wide association study. Among 17,893 traits, 29 traits were found to be significantly related to AAM, including younger age at the first sexual intercourse, younger age of starting oral contraceptives, younger age at first delivery, and an increased risk of childhood sexual abuse, all of which are known to increase the risk for depression. The authors failed to replicate selected findings, for example, regarding the risk for childhood sexual abuse in an independent sample, but the direction and effect size were consistent. Thus, many of the identified traits could be on a causal pathway originating from (genetically determined) AAM as downstream mechanisms. Thus, the exact mechanisms by which AAM affects depression have yet to be conclusively elucidated but are likely related to the downstream outcomes of AAM.

### 4.3 Limitations

The analyses in the present study accounted for BMI, but this relied on the GWAS by Pulit et al. (2019) conducted in adults and, thus, the assumption of continuity of its genetic architecture over the life course. A recent meta-analysis of 26 GWASs on childhood BMI, including the data from 61,111 children, identified 25 loci (Vogelezang et al., 2020). In line with our assumption, most of these SNPs (20 of 25) were also related to adult BMI, and there was a genetic correlation between childhood and adult BMI of *r*_*g*_ = 0.76. Moreover, the GWAS by Pulit et al. (2019) allowed us to use sex-specific BMI-related findings pertaining only to females.

The genetic instruments for EdAtt, WBS, and depression were not sex-specific, as no such information is available. However, sex was included as a covariate in the regression models of the GWASs of EdAtt and depression. Moreover, regarding depression, a recent study that evaluated between-sex genetic heterogeneity in MDD using GWAS summary statistics from 29 cohorts found a genetic correlation close to one (Trzaskowski et al., 2019), and an even more recent GWAS meta-analysis suggests that for a broad range of neuropsychiatric traits, including MDD, the effect of sex is only small and polygenic (Martin et al., 2021). Thus, the results of our analyses are unlikely to be affected by missing the gender-specific effect estimates.

We were limited to analyzing a linear relationship between AAM and depression as the estimation of nonlinear effects requires individual-level genetic data that were not available to us. However, a nonlinear relationship cannot be excluded. In a study by Day et al. (2015), earlier menarche was associated with a higher risk of all-cause mortality, while later menarche was not protective but likewise harmful. Interestingly, regarding depression, there was no evidence of any relationship, either linear or nonlinear, after correction for multiple testing. However, this may have been an issue of the (non-genetic) statistical approach chosen and/or insufficient power. Nonetheless, even when assuming a curvilinear relationship, this would support the conclusion that earlier AAM was causally related to increased depression risk, even though we could not consider nonlinear effects in the present study.

Two-sample MR assumes independent samples for the instrument variable and the outcome. Violation of this assumption may lead to inflated type 1 error and biased effect estimates. This, however, only applies to continuous but not binary outcomes, as in the present study (Burgess et al., 2016).

As previously mentioned, the covariance between AAM, BMI, EdAtt, and WBC could not be considered in the multivariable MR analysis because individual data were not available. Moreover, our results apply to the Caucasian population only and likely do not generalize to patients with precocious puberty, even though SNPs in genes related to precocious puberty were used as IV as well (Day et al., 2017).

### 4.4 Implications

The present study provides evidence for a causal contribution of an earlier AAM to a higher risk for depression, which has several implications. First, the results of the present study support conclusions from previous epidemiological studies implicating AAM in depression without the pitfalls of residual confounding and reverse causation. Second, as a consequence, health care providers should be aware of the timing of menarche in women and its potential lifelong implications for mental health. Thus, AAM could be considered a prognostic factor for a clinical risk score assessing the probability of depression during adolescence, also in terms of mental illness prevention, to help guide practitioners identify young women at risk for depression. Third, considering the adverse consequences of an earlier AAM on mental health, this should foster efforts to address risk factors (e.g., BMI, socioeconomic status, paternal absence, migration status, and birth weight) that promote an earlier AAM and/or to promote protective factors, including the duration of schooling and a higher level of educational attainment, to mitigate a genetically determined pathway to depression.

## Data Availability

Publicly available datasets analyzed in this study can be found here: https://www.ebi.ac.uk/gwas/. Original data generated and analyzed during this study (i.e., harmonized genetic data) can be found here: https://doi.org/10.6084/m9.figshare.19518982.v4.
