# Supplementary material for: Causal Effect of Age at Menarche on the Risk for Depression: Results From a Two-Sample Multivariable Mendelian Randomization Study
Source: Front Genet. 2022 Jul 12;13:918584. doi: 10.3389/fgene.2022.918584 (PMC9315288; doi:10.3389/fgene.2022.918584)
Supplement: Supplementary file 1 [file DataSheet1.docx]

Supplementary Material

**Evidence of a Causal Effect of Age at Menarche on the Risk for Depression: Results from a Two-Sample Multivariable Mendelian Randomization Study**

Raphael Hirtz^1,2^, Christine Hars^1^, Roaa Naaresh^2^, Björn‑Hergen Laabs^3^, Jochen Antel^2^, Corinna Grasemann^4^, Anke Hinney^2^, Johannes Hebebrand^2^, Triinu Peters^2^

**Outline**

Table S1 - Univariable MR: The effect of age at menarche on risk for depression

Table S2 - Univariable MR: The effect of age at menarche on risk for depression after exclusion of BMI-associated SNPs

Table S3 - Univariable MR: The effect of AAM on risk for depression (n=183 SNPs) after exclusion of SNPs associated with BMI, educational attainment and white blood count were excluded

Figure S1. Scatter plot of genetic associations with age at menarche against risk for depression using different Mendelian randomization methods

Figure S2. Funnel plot: Mendelian randomization analyses with age at menarche as exposure and risk for depression as outcome

Figure S3. Leave-one-out analyses using the IVW method: Mendelian randomization analyses with age at menarche as exposure and risk for depression as outcome

Figure S4. Power analysis

Figure S5. Scatter plot of genetic associations with age at menarche against risk for depression using different Mendelian randomization methods after exclusion of BMI-associated SNPs

Figure S6. Funnel plot: Mendelian randomization analyses with age at menarche as exposure and risk for depression as outcome after exclusion of BMI-associated SNPs

Figure S7. Leave out analyses using the IVW method: Mendelian randomization analyses with age at menarche as exposure and risk for depression as outcome after exclusion of BMI-associated SNPs

Figure S8. Scatter plot of genetic associations with age at menarche against risk for depression using different Mendelian randomization methods after exclusion of SNPs associated with BMI, educational attainment, and/or white blood count

Figure S9 Funnel plot: Mendelian randomization analyses with age at menarche as exposure and risk for depression as outcome after exclusion of SNPs associated with BMI, educational attainment, and/or white blood count

Figure S10. Leave-one-out analyses using IVW method: Mendelian randomization (MR) analyses with age at menarche (AAM) as exposure and risk for depression as outcome after exclusion of SNPs associated with BMI, educational attainment, and/or white blood count

Supplementary References

**Univariable MR: The effect of AAM on risk for depression (all SNPs n=343)**

Table S1. Results of MR analyses regarding the overall causal effect of age at menarche (AAM) on the risk for depression calculated using different Mendelian randomization (MR) methods.

| Method | b | | | SE | P-value ^a^ | OR | | |
| --- | --- | --- | --- | --- | --- | --- | --- | --- |
|  | Point estimate | Lower 95% CI | Upper 95% CI |  |  | Point estimate | Lower 95% CI | Upper 95% CI |
| Inverse variance weighted | -0.042 | -0.065 | -0.019 | 0.012 | **3.2 x 10^-04^** | 0.959 | 0.937 | 0.981 |
| MR Egger | -0.047 | -0.108 | 0.014 | 0.031 | 0.130 | 0.954 | 0.898 | 1.014 |
| MR Egger (bootstrap) | -0.080 | -0.120 | -0.041 | 0.020 | **3.0 x 10^-05^** | 0.923 | 0.888 | 0.960 |
| Simple mode | -0.046 | -0.135 | 0.043 | 0.045 | 0.308 | 0.955 | 0.874 | 1.044 |
| Weighted mode | -0.065 | -0.114 | -0.015 | 0.025 | **0.011** | 0.937 | 0.892 | 0.985 |
| Simple median | -0.043 | -0.067 | -0.019 | 0.012 | **4.50 x 10^-04^** | 0.958 | 0.935 | 0.981 |
| Weighted median | -0.048 | -0.075 | -0.022 | 0.013 | **2.71 x 10^-04^** | 0.953 | 0.928 | 0.978 |
| MR RAPS | -0.042 | -0.065 | -0.020 | 0.012 | **2.71 x 10^-04^** | 0.959 | 0.937 | 0.981 |
| MR PRESSO (outlier corrected) | -0.036 | -0.057 | -0.015 | 0.011 | **8.57 x 10^-04^** | 0.965 | 0.944 | 0.985 |
| Methods robust for heterogeneity | | |  |  |  |  |  |  |
| Penalised weighted median | -0.053 | -0.078 | -0.028 | 0.013 | **3.29 x 10^-05^** | 0.948 | 0.925 | 0.972 |
| MR Lasso ^b^ | -0.051 | -0.068 | -0.035 | 0.009 | **1.84 x 10^-09^** | 0.950 | 0.934 | 0.967 |
| Contamination mixture method ^c^ | -0.090 | -0.110 | -0.070 |  | **9.50 x 10^-07^** | 0.914 | 0.896 | 0.932 |

b = unstandardized causal estimate of the change in risk for depression per one-year change in age of menarche, OR = odds ratio, SE = standard error, CI = confidence interval; ^a^ significant findings are printed in bold type; ^b^ number of valid instruments: 262, ^c^ standard deviation of invalid estimates = 0.371; tuning parameter = 0.0985.

**Univariable MR: The effect of AAM on risk for depression. SNPs associated with BMI were excluded (n=263)**

Table S2. Results of MR analyses of the overall causal effect of age at menarche (AAM) on the risk for depression calculated using different Mendelian randomization (MR) methods. SNPs associated with BMI (F>10) were excluded (N=263).

| Method | b | | | SE | P-value ^a^ | OR | | |
| --- | --- | --- | --- | --- | --- | --- | --- | --- |
|  | Point estimate | Lower 95% CI | Upper 95% CI |  |  | Point estimate | Lower 95% CI | Upper 95% CI |
| Inverse variance weighted | -0.031 | -0.056 | -0.006 | 0.013 | **0.014** | 0.969 | 0.946 | 0.994 |
| MR Egger | -0.046 | -0.113 | 0.021 | 0.034 | 0.182 | 0.955 | 0.893 | 1.022 |
| MR Egger (bootstrap) | -0.056 | -0.102 | -0.011 | 0.023 | **0.008** | 0.945 | 0.903 | 0.989 |
| Simple mode | -0.036 | -0.129 | 0.058 | 0.048 | 0.454 | 0.965 | 0.879 | 1.059 |
| Weighted mode | -0.086 | -0.145 | -0.027 | 0.030 | **0.005** | 0.918 | 0.865 | 0.974 |
| Simple median | -0.035 | -0.064 | -0.007 | 0.015 | **0.015** | 0.965 | 0.938 | 0.993 |
| Weighted median | -0.049 | -0.078 | -0.020 | 0.015 | **0.001** | 0.952 | 0.925 | 0.980 |
| MR RAPS | -0.037 | -0.062 | -0.012 | 0.013 | **0.004** | 0.964 | 0.940 | 0.988 |
| MR PRESSO (outlier corrected) | -0.033 | -0.057 | -0.009 | 0.012 | **0.008** | 0.968 | 0.945 | 0.991 |
| Methods robust for heterogeneity | | | | | | | | |
| Penalised weighted median | -0.054 | -0.082 | -0.027 | 0.014 | **1.2 x 10^-04^** | 0.947 | 0.921 | 0.974 |
| MR Lasso ^b^ | -0.050 | -0.070 | -0.031 | 0.010 | **4.8 x 10^-07^** | 0.951 | 0.932 | 0.970 |
| Contamination mixture method ^c^ | -0.088 | -0.11 | -0.06 |  | **7.1 x 10^-05^** | 0.916 | 0.896 | 0.936 |

b = unstandardized causal estimate of the change in risk for depression per one-year change in age of menarche, OR = odds ratio, SE = standard error, CI = confidence interval; ^a^ significant findings are printed in bold type; ^b^ number of valid instruments: 207, ^c^ standard deviation of invalid estimates = 0.351; tuning parameter = 0.1158.

**Univariable MR: The effect of AAM on risk for depression (n=183 SNPs). SNPs for BMI, educational attainment and white blood count were excluded**

Table S3. Results of the Mendelian randomization (MR) analyses regarding the overall causal effect of age at menarche (AAM) on the risk for depression calculated using different MR methods. SNPs associated with BMI, educational attainment, and/or white blood count (F>10) were excluded (N=185).

| Method | b | | | SE | P-value ^a^ | OR | | |
| --- | --- | --- | --- | --- | --- | --- | --- | --- |
|  | Point estimate | Lower 95% CI | Upper 95% CI |  |  | Point estimate | Lower 95% CI | Upper 95% CI |
| MR Egger | -0.040 | -0.125 | 0.045 | 0.043 | 0.355 | 0.961 | 0.882 | 1.046 |
| MR Egger (bootstrap) | -0.032 | -0.093 | 0.029 | 0.031 | 0.153 | 0.969 | 0.912 | 1.030 |
| Inverse variance weighted | -0.041 | -0.070 | -0.013 | 0.015 | **0.005** | 0.960 | 0.933 | 0.987 |
| Simple median | -0.048 | -0.082 | -0.015 | 0.017 | **0.004** | 0.953 | 0.922 | 0.985 |
| Weighted median | -0.050 | -0.082 | -0.017 | 0.017 | **0.003** | 0.951 | 0.921 | 0.983 |
| Simple mode | -0.042 | -0.147 | 0.063 | 0.054 | 0.443 | 0.959 | 0.863 | 1.065 |
| Weighted mode | -0.052 | -0.141 | 0.037 | 0.045 | 0.255 | 0.949 | 0.869 | 1.038 |
| Robust adjusted profile score (RAPS) | -0.045 | -0.074 | -0.015 | 0.015 | **0.003** | 0.956 | 0.929 | 0.985 |
| MR PRESSO (outlier corrected) | -0.039 | -0.066 | -0.011 | 0.014 | **0.005** | 0,962 | 0.936 | 0.989 |
| Methods robust for heterogeneity | | | | | | | | |
| Penalized weighted median | -0.055 | -0.090 | -0.021 | 0.018 | **0.002** | 0.946 | 0.914 | 0.979 |
| MR-Lasso ^b^ | -0.057 | -0.080 | -0.035 | 0.012 | **7.6 x 10^-07^** | 0.945 | 0.923 | 0.881 |
| Contamination mixture method ^c^ | -0.088 | -0.127 | -0.048 |  | **5.5 x 10^-04^** | 0.916 | 0.881 | 0.952 |

b = unstandardized estimate of the change in risk for depression per one-year change in age of menarche, OR = odds ratio, SE = standard error, CI = confidence interval. ^a^ significant findings are printed in bold type; ^b^ number of variants: 185; number of valid instruments: 152, ^c^ standard deviation of invalid estimates = 0.334; tuning parameter = 0.1417.


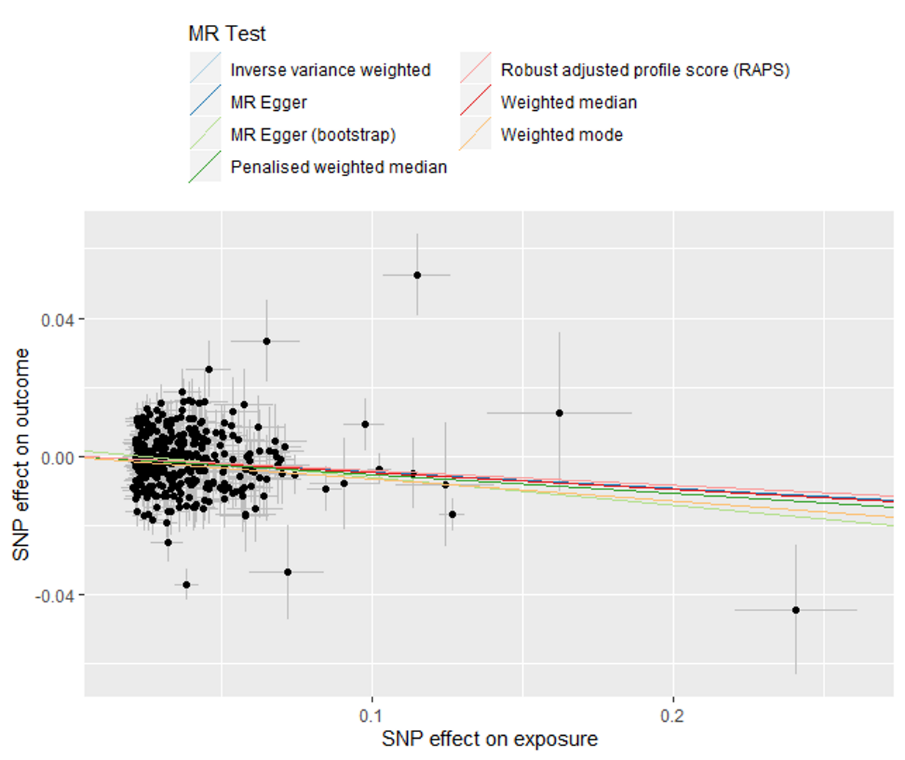


Figure S1. Scatter plot of genetic associations with age at menarche (AAM) against risk for depression using different Mendelian randomization (MR) methods. The slopes of each line represent the causal association for each method.


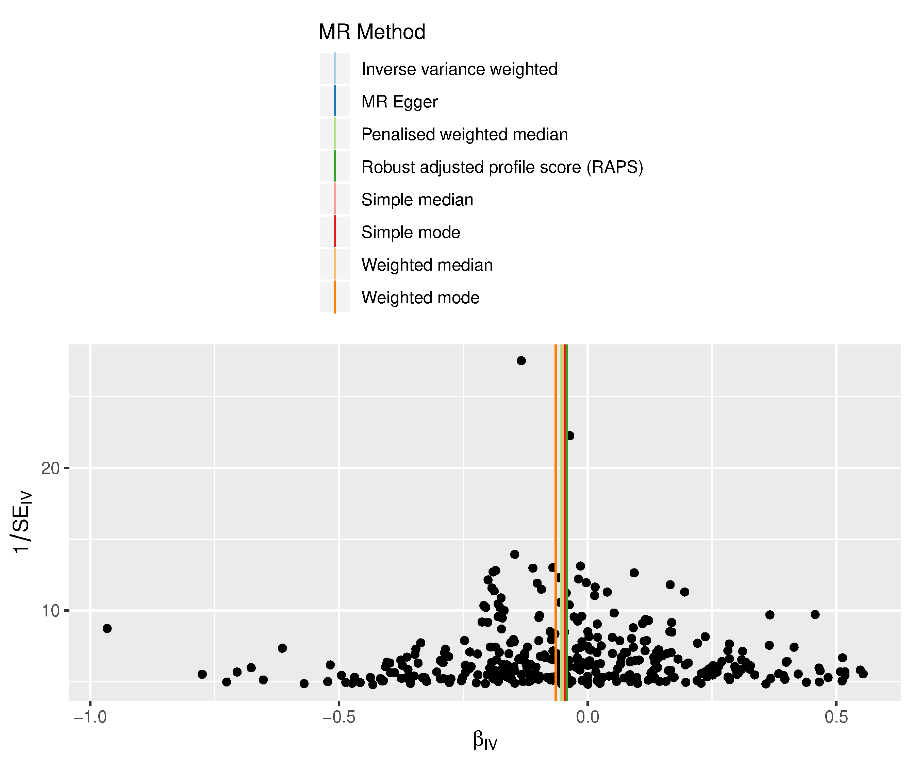


Figure S2. Funnel plot: Mendelian randomization (MR) analyses with age at menarche (AAM) as exposure and risk for depression as outcome. SE = standard error, IV = instrumental variable, β = unstandardized causal estimate of the change in risk for depression per one-year change in age of menarche.


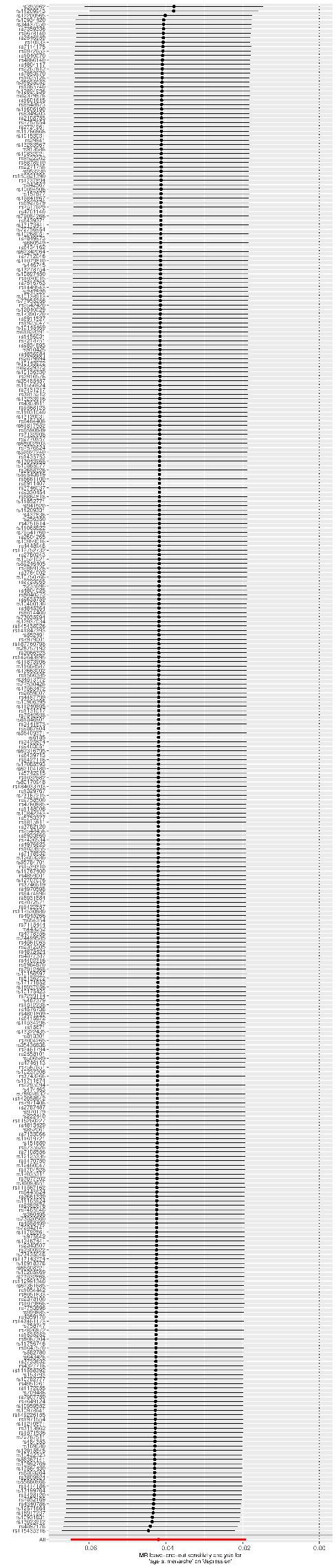


Figure S3. Leave-one-out analyses using the IVW method: Mendelian randomization (MR) analyses with age at menarche (AAM) as exposure and risk for depression as outcome.


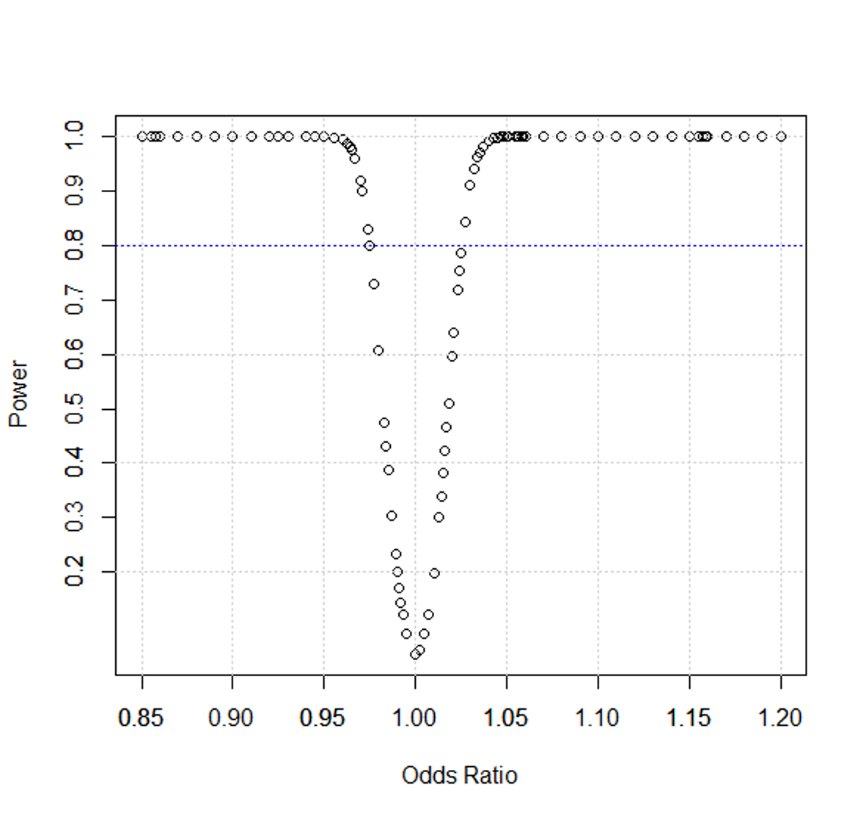


Figure S4. Calculated power to detect a true causal effect: power of 0.80 for OR= 0.975/1.026; the following data were used for the calculation according to Brion et al. (2013): K = 0.305 (depression), N = 807,553 (depression), R^2^_xy_ = 0.072 (age at menarche).


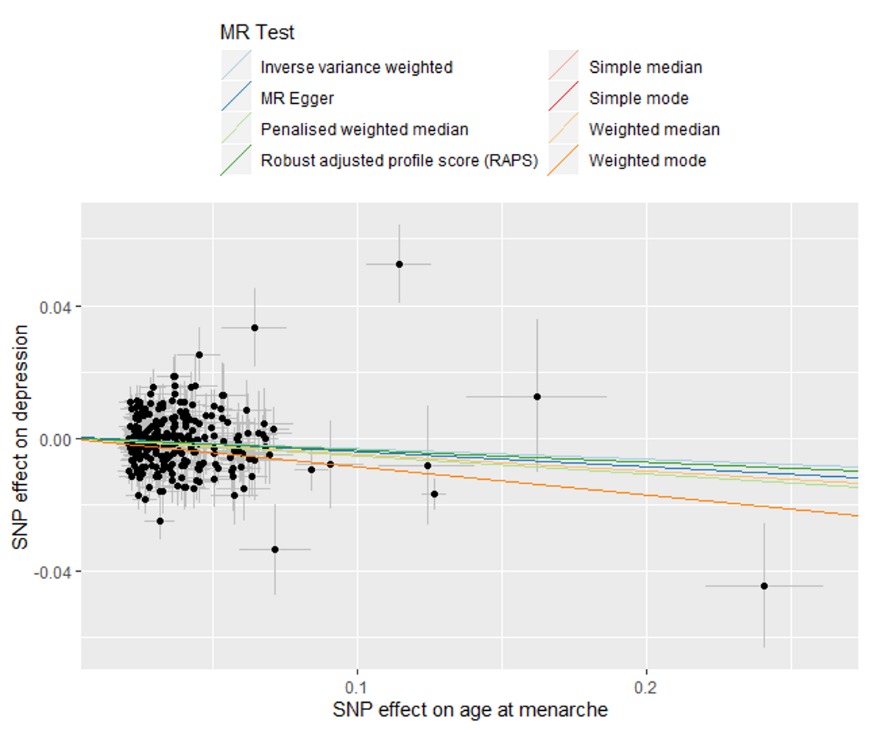


Figure S5. Scatter plot of genetic associations with age at menarche (AAM) against risk for depression using different Mendelian randomization (MR) methods. SNPs associated with BMI (F>10) were excluded. The slopes of each line represent the causal association for each method.


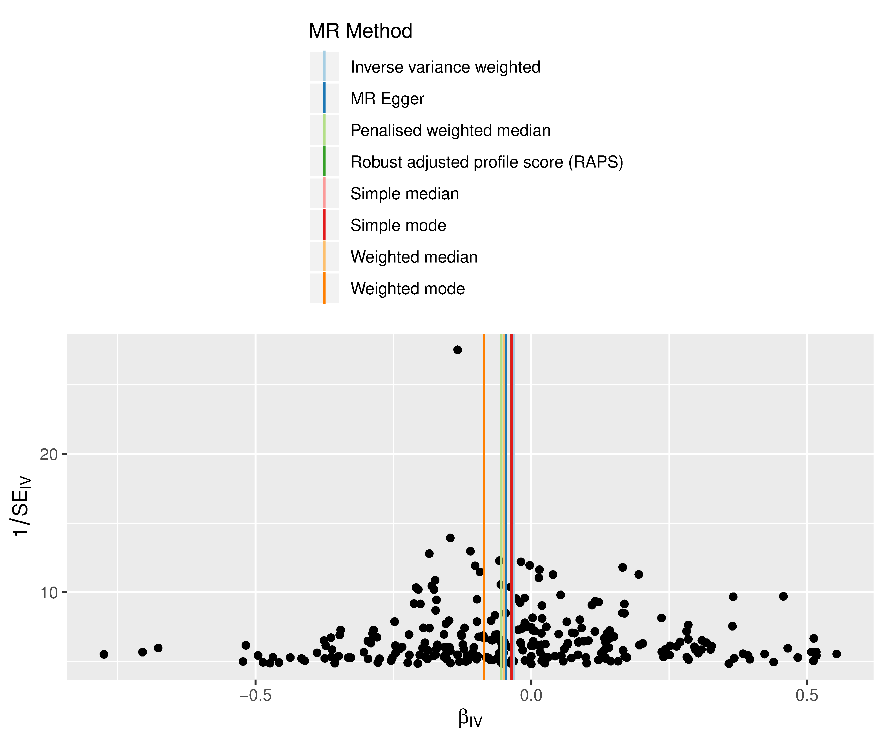


Figure S6. Funnel plot: Mendelian randomization (MR) analyses with age at menarche (AAM) as exposure and risk for depression as outcome. SNPs associated with BMI (F>10) were excluded. SE = standard error, IV = instrumental variable, β = unstandardized causal estimate of the change in risk for depression per one-year change in age of menarche.


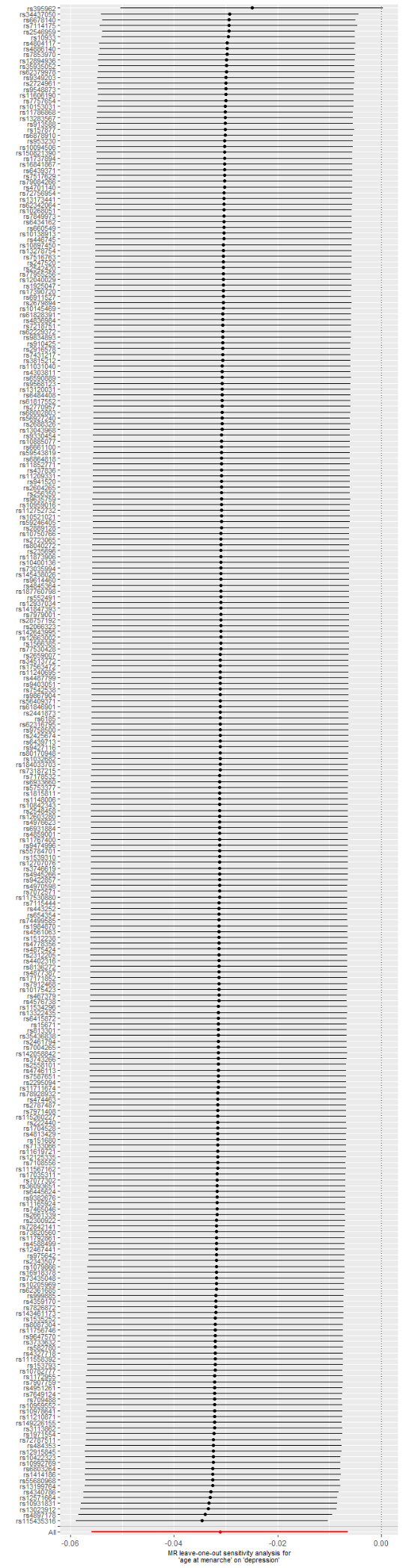


Figure S7. Leave out analyses using the IVW method: Mendelian randomization (MR) analyses with age at menarche (AAM) as exposure and risk for depression as outcome. SNPs associated with BMI (F>10) were excluded.


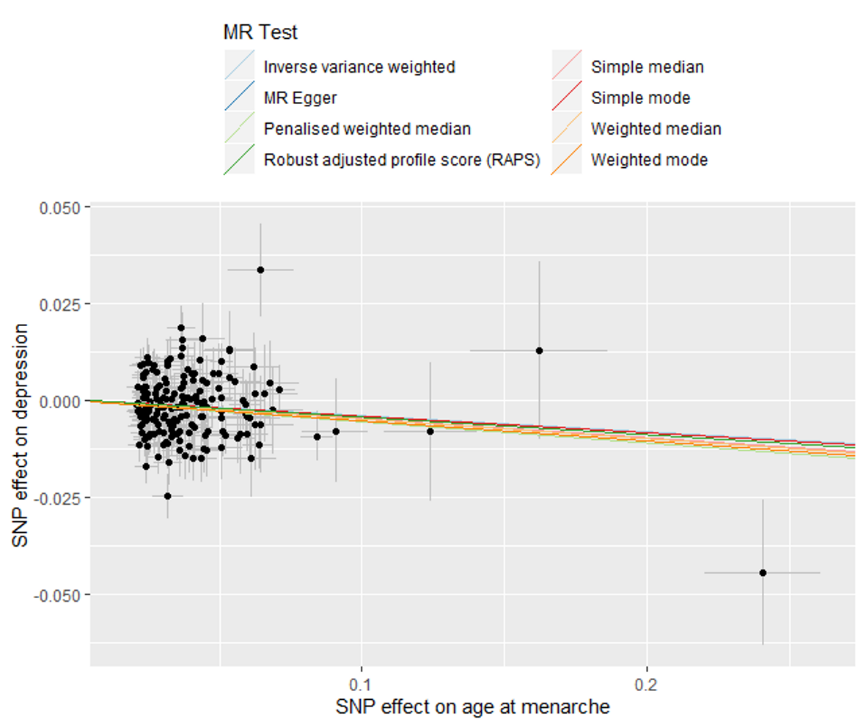


Figure S8. Scatter plot of genetic associations with age at menarche (AAM) against risk for depression using different Mendelian randomization (MR) methods. SNPs associated with BMI, educational attainment, and/or white blood count (F>10) were excluded. The slopes of each line represent the causal association for each method.


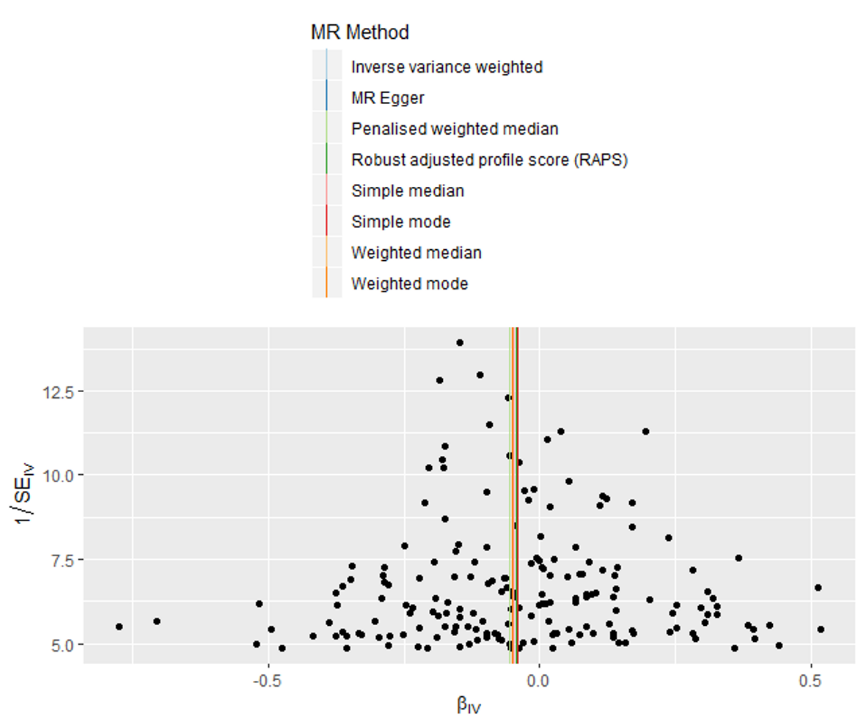


Figure S9 Funnel plot: Mendelian randomization (MR) analyses with age at menarche (AAM) as exposure and risk for depression as outcome. SNPs associated with BMI, educational attainment, and/or white blood count (F>10) were excluded. SE = standard error, IV = instrumental variable, β = unstandardized causal estimate of the change in risk for depression per one-year change in age of menarche


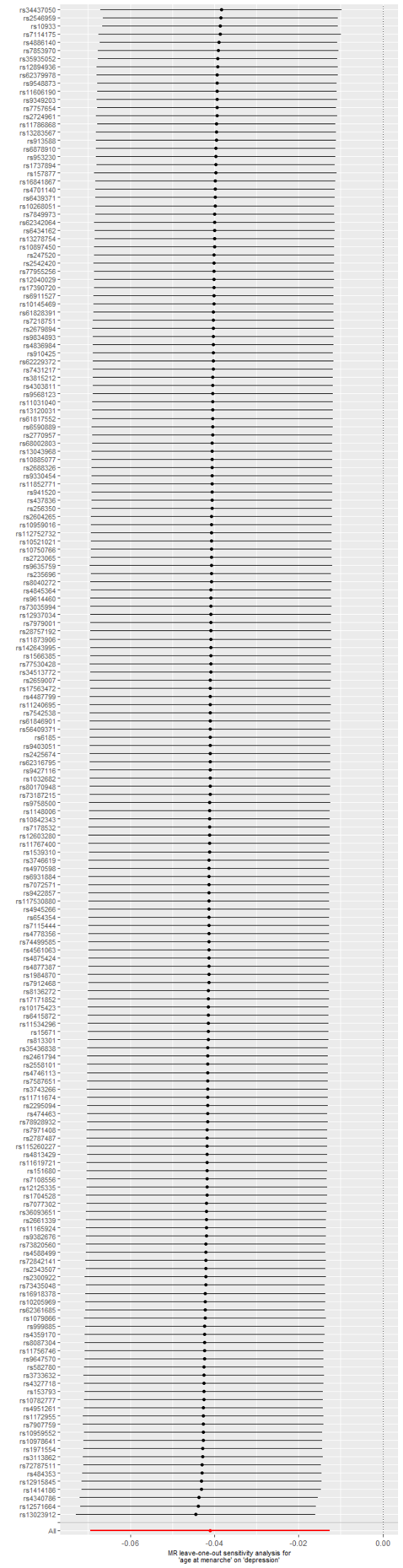


Figure S10. Leave-one-out analyses using IVW method: Mendelian randomization (MR) analyses with age at menarche (AAM) as exposure and risk for depression as outcome. SNPs associated with BMI, educational attainment, and/or white blood count (F>10) were excluded.

**References**

Brion, M.J., Shakhbazov, K., and Visscher, P.M. (2013). Calculating statistical power in Mendelian randomization studies. *Int J Epidemiol* 42(5)**,** 1497-1501. doi: 10.1093/ije/dyt179.
